# EHMTI-0363. Quality of life in subjects treated by non-invasive vagus nerve stimulation using gammacore® for the prevention and acute treatment of chronic cluster headache

**DOI:** 10.1186/1129-2377-15-S1-I6

**Published:** 2014-09-18

**Authors:** C Gaul, H Diener, K Solbach, N Silver, A Straube, D Magis, U Reuter, A Andersson, EJ Liebler

**Affiliations:** 1 Migraine and Headache Clinic, University of Duisburg-Essen, Königstein, Germany; 2 Department of Neurology, University Hospital Essen, Essen, Germany; 3 Department of Neurology, Walton Centre for Neurology and Neurosurgery, Liverpool, UK; 4 Department of Neurology, Ludwig Maximilian University of Munich, Munich, Germany; 5 Department of Neurology, Liège University, Liège, Belgium; 6 Berlin NeuroImaging Center, Charité University Hospital, Berlin, Germany; 7 Clinical Affairs, electroCore Medical, Gothenburg, Sweden; 8 Scientific Medical and Clinical Affairs, electroCore LLC, Basking Ridge, USA

## Introduction

The debilitating nature of chronic cluster headache (CH) can negatively impact a patient's quality of life (QoL). In recent years, non-invasive neuromodulation devices have been of increasing interest for the treatment of CH.

## Aim

The Prevention and Acute Treatment of Chronic Cluster Headache (PREVA) study was designed to evaluate the clinical effects—including QoL outcomes—of gammaCore®, a non-invasive vagus nerve stimulation device (nVNS), in subjects with chronic CH.

## Methods

PREVA was a randomized, well-controlled study comprised of a 2-week run-in phase, a 4-week randomized (1:1; nVNS vs standard of care [SoC]) phase, and a 4-week extension phase. Subjects delivered stimulations prophylactically twice daily (mandatory) or optionally for the rescue treatment of CH attacks. Three validated scales (EQ-5D-3L™, Headache Impact Test™ [HIT-6], and Hospital Anxiety and Depression Scale [HADS]) were used to assess the QoL of subjects at the end of each study phase.

## Results

A total of 97 subjects were randomized to treatment; data from 93 subjects (n=45 nVNS; n=48 SoC) were included in the efficacy analysis population. Compared with subjects treated with SoC alone, subjects also treated with nVNS reported greater overall improvements in EQ-5D-3L, HIT-6, and HADS scores from the end of run-in to the end of the randomized phase. As of June 2014, QoL data from the extension phase were not available; however, they will be available for presentation at EHMTIC.

## Conclusion

Compared with SoC, use of nVNS for the both preventive and acute treatment of CH was associated with greater improvement in QoL as assessed on 3 validated instruments.

Abstract submitted on behalf of the PREVA Study Investigators.

